# Pregnancy Outcomes, Immunophenotyping and Immunohistochemical Findings in a Cohort of Pregnant Patients with COVID-19—A Prospective Study

**DOI:** 10.3390/diagnostics13071345

**Published:** 2023-04-04

**Authors:** Ana-Maria Adam, Radu-Florin Popa, Cristian Vaduva, Costinela Valerica Georgescu, Gigi Adam, Alina-Sinziana Melinte-Popescu, Cristina Popa, Demetra Socolov, Aurel Nechita, Ingrid-Andrada Vasilache, Elena Mihalceanu, AnaMaria Harabor, Marian Melinte-Popescu, Valeriu Harabor, Anca Neagu, Razvan Socolov

**Affiliations:** 1 Clinical and Surgical Department, Faculty of Medicine and Pharmacy, ‘Dunarea de Jos’ University, 800216 Galati, Romania; 2 Department of Vascular Surgery, University of Medicine and Pharmacy “Grigore T. Popa”, 700111 Iasi, Romania; 3 Department of Mother and Child Medicine, Faculty of Medicine, University of Medicine and Pharmacy, 200349 Craiova, Romania; 4 Department of Pharmaceutical Sciences, Faculty of Medicine and Pharmacy, Dunarea de Jos University, 800216 Galati, Romania; 5 Department of Mother and Newborn Care, Faculty of Medicine and Biological Sciences, ‘Ștefan cel Mare’ University, 720229 Suceava, Romania; 6 Discipline of Oral Medicine, ‘Grigore T. Popa’ University of Medicine and Pharmacy, 700115 Iasi, Romania; 7 Department of Obstetrics and Gynecology, ‘Grigore T. Popa’ University of Medicine and Pharmacy, 700115 Iasi, Romania; 8 Department of Internal Medicine, Faculty of Medicine and Biological Sciences, ‘Ștefan cel Mare’ University, 720229 Suceava, Romania; 9 ‘Saint John’ Clinical Emergency Hospital for Children, 800487 Galati, Romania

**Keywords:** COVID-19, SARS-CoV-2, pregnancy outcomes, immunophenotyping, immunohistochemistry

## Abstract

(1) Background: SARS-CoV-2 infection during pregnancy could determine important maternal and fetal complications. We aimed to prospectively assess placental immunohistochemical changes, immunophenotyping alterations, and pregnancy outcomes in a cohort of patients with COVID-19; (2) Methods: 52 pregnant patients admitted to a tertiary maternity center between October 2020 and November 2021 were segregated into two equal groups, depending on the presence of SARS-CoV-2 infection. Blood samples, fragments of umbilical cord, amniotic membranes, and placental along with clinical data were collected. Descriptive statistics and a conditional logistic regression model were used for data analysis; (3) Results: Adverse pregnancy outcomes such as preterm labor and neonatal intensive care unit admission did not significantly differ between groups. The immunophenotyping analysis indicated that patients with moderate–severe forms of COVID-19 had a significantly reduced population of T lymphocytes, CD4+ T cells, CD8+ T cells (only numeric), CD4+/CD8+ index, B lymphocytes, and natural killer (NK) cells. Our immunohistochemistry analysis of tissue samples failed to demonstrate positivity for CD19, CD3, CD4, CD8, and CD56 markers; (4) Conclusions: Immunophenotyping analysis could be useful for risk stratification of pregnant patients, while further studies are needed to determine the extent of immunological decidual response in patients with various forms of COVID-19.

## 1. Introduction

Severe acute respiratory syndrome coronavirus 2 (SARS-CoV-2) is the causative agent of coronavirus disease 2019 (COVID-19) [1]. Since the World Health Organization (WHO) declared the SARS-CoV-2 a pandemic on March 11, 2020, new questions have emerged as to whether this virus can affect pregnancy outcomes [2]. Many studies have estimated that between 3 and 20% of pregnant women presenting for labor and delivery were infected with this virus [3,4]; however, because universal screening was not widely used, it is challenging to offer an exact estimate for the prevalence of this viral infection in the population of pregnant patients.

Until now, several studies have outlined an association between SARS-CoV-2 infection and poor pregnancy outcomes [5]. For example, it was demonstrated that miscarriage, intrauterine death, fetal growth restriction, and high maternal–fetal morbidity rates were more prevalent in patients infected with this type of virus [6,7,8,9,10]. Wei et al. performed a meta-analysis on 42 studies involving 438 548 pregnant patients, which evaluated the association between SARS-CoV-2 infection during pregnancy and adverse pregnancy outcomes. The authors demonstrated that the risk of developing preeclampsia (OR 1.33, 95% CI 1.03 to 1.73), preterm birth (OR 1.82, 95% CI 1.38 to 2.39), and stillbirth (OR 2.11, 95% CI 1.14 to 3.90) were higher in infected patients, and that severe forms of viral infection were associated with higher odds of developing preeclampsia, preterm birth, gestational diabetes, or delivering a newborn with low birth weight [11].

The risk of maternal complications appears to be increased in severe forms of COVID-19. A retrospective cohort study by Ko et al. on 489 471 delivery hospitalizations evaluated the maternal complications for 6550 pregnant patients with COVID-19 [12]. The authors demonstrated that this viral infection was associated with increased risk for acute respiratory distress syndrome, death, sepsis, mechanical ventilation, shock, intensive care unit admission, acute renal failure, thromboembolic disease, and adverse cardiac events. The risk factors for severe COVID-19 disease are poorly explored in the literature, but recent data suggest that maternal diabetes, obesity, asthma, lower respiratory symptoms, and the extent of pulmonary disease on imaging studies constitute important independent risk factors for this maternal intensive care admission [9,13,14,15,16].

On the other hand, it was shown that the prevalence of adverse pregnancy outcomes was reduced in vaccinated patients. For example, a recent meta-analysis by Prasad et al. indicated that messenger ribonucleic acid (mRNA) vaccination was a safe and effective method for reducing the rate of stillbirth by 15% in pregnant patients with confirmed SARS-CoV-2 infection (pooled odds ratio-OR: 0.85; 95% confidence interval—CI: 0.73–0.99, 66,067 vaccinated vs. 424,624 unvaccinated, I^2^ = 93.9%) [17]. The safety of mRNA vaccines has been outlined by many observational studies that compared perinatal outcomes between vaccinated and unvaccinated pregnant patients and did not demonstrate harmful effects on pregnancy or the newborn [18,19,20].

There are ongoing efforts to investigate placentas from COVID-19 patients in order to better comprehend and predict the consequences of SARS-CoV-2 on pregnant women and newborns. The placenta acts as a barrier, preventing the fetus from being exposed to maternal illnesses [21]. On the other hand, the receptor angiotensin-converting enzyme 2 (ACE2), used by SARS-CoV-2 for infecting pulmonary cells, was also found in placental tissue [22,23,24], supporting the idea of vertical transmission for this type of infection [25,26]. Recent investigations of placental pathologies indicated the presence of feto-maternal vascular malperfusion and placental inflammation with excessive infiltration of CD3+ CD8+ lymphocytes, CD68+ macrophages, and CD20+ lymphocytes [27,28,29].

Given the scarcity of data on placental changes in the context of SARS-CoV-2 infection, the goal of this study was to assess the immunohistochemical changes in the umbilical cord, amniotic membranes, and placental fragments, the immunophenotyping alterations, as well as pregnancy outcomes, in a cohort of patients with this type of viral infection.

## 2. Materials and Methods

We conducted an observational prospective study of pregnant patients with COVID-19 admitted to the Obstetrics and Gynecology Hospital ‘Buna Vestire’, Galati, between October 2020 and November 2021. The SARS-CoV-2 infection was confirmed after the evaluation of nasopharyngeal swabs using the polymerase chain reaction (PCR) assay. The main circulating variants of SARS-CoV-2 were Alpha, for October 2020–September 2021 timeframe, and Delta, for October–November 2021 timeframe [30,31].

Ethical approval for this study was obtained from the Institutional Ethics Committees of University of Medicine and Pharmacy ‘Grigore T. Popa’ (No.27/04.01.2021), and ‘Buna Vestire’ Obstetrics and Gynecology Hospital (No. 6793/08.09.2020). Informed consent was obtained from all participants included in the study. All methods were carried out in accordance with relevant guidelines and regulations.

Exclusion criteria comprised patients who had multiple pregnancies, ectopic pregnancies, first and second trimester abortions, fetal intrauterine demise, fetuses with chromosomal or structural abnormalities, incomplete medical records, or who were unable to offer informed consent due to various reasons (age less than 18 years old, intellectual deficits, psychiatric disorders, etc.).

A total of 52 patients were segregated into two groups depending on the presence of SARS-CoV-2 infection: with confirmed infection (*n* = 26) or without infection (*n* = 26). The following variables were recorded: demographic data, the patient’s medical history, clinical manifestations, laboratory parameters at admission, pregnancy outcomes, imaging findings, the treatment received, their clinical evolution, and the neonatal outcomes (gestational age, birthweight, Apgar score at 5 min).

For investigating the immunophenotyping changes, we further segregated the group of patients with COVID-19 into two subgroups depending on the disease severity: mild disease (subgroup 1, *n* = 14 patients), and moderate–severe disease (subgroup 2, *n* = 12 patients). The criteria for inclusion in the mild disease subgroup were derived from the National Guidelines [32], and were represented by patients with confirmed SARS-CoV-2 infection, with any signs or symptoms of respiratory tract infection, such as fever, cough, rhinorrhea, sore throat, malaise, headache, or myalgia, but without breathing difficulties, dyspnea, or thoracic imaging findings suggestive of pneumonia.

On the other hand, patients with moderate or severe forms of COVID-19 were considered in the case of confirmed SARS-CoV-2 infection, fever and signs of non-severe pneumonia, without needing supplementary oxygen or who manifested important dyspnea (respiratory frequency ≥ 30/min), newly onset hypoxemia (oxygen saturation ≤ 94%), a ratio of arterial oxygen partial pressure (mmHg) to fractional inspired oxygen (PaO_2_/FiO_2_) ≤300 mmHg, rapid evolution of pulmonary imaging in the last 24–48 h (>50%), a progressive reduction in the lymphocytes number, or a rapid increase in serum lactate levels.

A blood sample (5 mL) was drawn from pregnant patients diagnosed with COVID-19 in tubes containing ethylenediaminetetraacetic acid (EDTA). The frequency and number of CD4+ T cells, CD8+ T cells, CD19+ B cells, CD16+ or CD56+, and NK cells were measured by flow cytometry method using human monoclonal antibodies (BD Biosciences), according to the manufacturer’s instructions. Flow cytometric acquisition was performed on a Partec PAS flow cytometer system (Partec GmBH), and the results were analyzed using Flowjo software (Treestar).

After delivery, two fragments of umbilical cord, amniotic membranes, and placenta underwent fixation in 10% buffered formalin and histopathological examination in the Pathology Department. Sections underwent routine processing, embedding, sectioning at 4 µm and staining with hematoxylin and eosin (H&E). For each formalin-fixed paraffin-embedded (FFPE) tissue sample, five additional 4 µm sections were collected for immunohistochemical (IHC) assays, which were performed on the DAKO Autostainer LINK 48. IHC antigen retrieval was performed using Target retrieval solution high pH (prediluted, pH 9.0) for 20 min at 97 °C. Specimens were incubated at room temperature as follows: for 5 min with Peroxidase blocking reagent, then with primary antibodies for 20 min, followed by Horseradish peroxidase for 20 min, then with DAB chromogen for 10 min, and they were then counterstained with hematoxylin. The primary antibodies (CD19, CD3, CD4, CD8, and CD56) were monoclonal, prediluted, ready to use (RTU), produced by Agilent, and their characteristics are mentioned in Table 1.

In the first stage of the statistical analysis, each variable was evaluated with chi-squared and Fisher’s exact tests for categorical variables, which were presented as frequencies with corresponding percentages, and t-tests for continuous variables, which were presented as means and standard deviations (SD).

A conditional logistic regression (CLR) model was applied for the evaluation of associations between adverse pregnancy outcomes and the presence of SARS-CoV-2 infection and the adjusted odd ratios (aOR) with 95% confidence intervals (CI) were calculated for each variable of interest. A *p* value less than 0.05 was considered statistically significant. The statistical analyses were performed using STATA SE (version 17, 2022, StataCorp LLC, College Station, TX, USA).

In the second stage of the analysis, we described the immunohistochemical changes in the umbilical cord, amniotic membranes, and placental fragments for pregnant patients infected with SARS-CoV-2.

## 3. Results

This prospective study evaluated 52 pregnant patients, segregated into two groups: with COVID-19 (group 1, *n* = 26 patients) and without COVID-19 (group 2, *n* = 26 patients). The clinical characteristics of the evaluated groups and the results from the univariate analysis are presented in Table 2. We found a statistically significant difference between the two groups regarding personal history of pulmonary disease (*p* = 0.038) and smoking habit (*p* = 0.037). Only one patient in the COVID-19 group had type II diabetes, and no other autoimmune disorders were identified in this cohort of patients. Even though we could not find a significant difference between groups regarding their body mass index (BMI), we noted a median value of 30 kg/m^2^ for this index, meaning that most pregnant patients had increased body weight.

For patients infected with COVID-19, the most prevalent symptoms were anosmia and cough (*n* = 7, 26.9%), ageusia (*n* = 6, 23.1%), fever (*n* = 5, 19.2%), followed by dyspnea (*n* = 4, 15.4%), myalgia and joint pain (*n* = 3, 11.5%), and diarrhea (*n* = 1, 3.8%). In two cases (7.7%), the imaging examinations revealed aspects of interstitial pneumonia. The univariate analysis of the main laboratory parameters revealed a significantly higher frequency of inflammatory syndrome (elevated C-reactive protein, procalcitonin, and ferritin) in the group of patients diagnosed with COVID-19 compared with controls (*p* < 0.05) (Table 3).

Our results failed to indicate a statistically significant difference between the two groups regarding the pregnancy outcomes: gestational age at birth (*p* = 0.42), birth weight (*p* = 0.39), mode of delivery (*p* = 0.56), preterm labor before 37 weeks of gestation (*p* = 0.22), and neonatal intensive care unit admission (*p* = 0.31), respectively (Table 4). Only Apgar score 5 min was significantly lower for the first group (*p* = 0.005), and patients with COVID-19 had a higher chance of delivering a newborn with a lower Apgar score (OR: 4.11; 95%CI: 1.211–13.974). All newborns were tested for SARS-CoV-2 infection, but none of them became positive during admission.

All patients with moderate–severe forms of COVID-19 received treatment with an antiviral (Remdesivir), thromboprophylaxis with low molecular weight heparin (LMWH), and antibiotic therapy. One maternal death was recorded due to interstitial pneumonia with acute respiratory distress syndrome and multiorgan system failure. None of the pregnant patients were vaccinated against COVID-19.

The pregnant patients with COVID-19 were segregated into two subgroups depending on the form of disease: mild disease (subgroup 1, *n* = 14 patients) and moderate–severe disease (subgroup 2, *n* = 12 patients). We compared the lymphocyte populations as determined by immunophenotyping between subgroups using univariate analysis, which is presented in Table 5. Our results indicated that patients with moderate–severe forms of COVID-19 had a significantly reduced population of lymphocytes, CD4+ T cells, CD8+ T cells (only numeric), and CD4+/CD8+ index compared with patients who presented with mild forms of COVID-19 (*p* < 0.05). Additionally, the populations of B lymphocytes and natural killer (NK) cells were significantly diminished in the first subgroup (*p* < 0.05).

**Figure 1:**
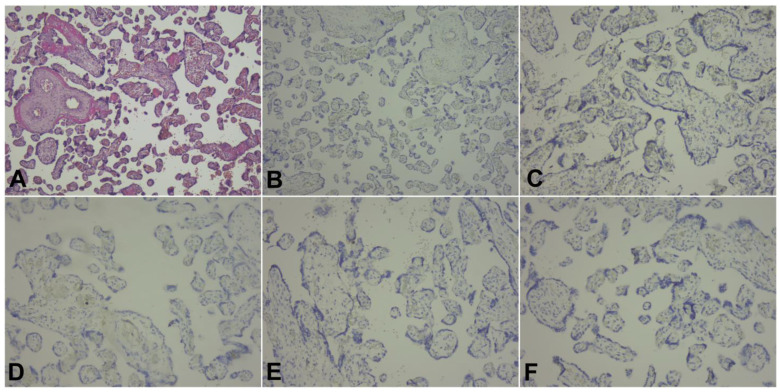
Selected microscopic aspects from placental sections: (**A**) Presence of intramural fibrin deposition and lack of inflammatory cells, H&E stain, ×100 magnification. (**B**–**F**) Negativity for the immune markings: (**B**) CD4, ×100 magnification, (**C**) CD8, ×200 magnification, (**D**) CD19, ×200 magnification, (**E**) CD3, ×200 magnification, (**F**) CD56, ×200 magnification.

**Figure 2:**
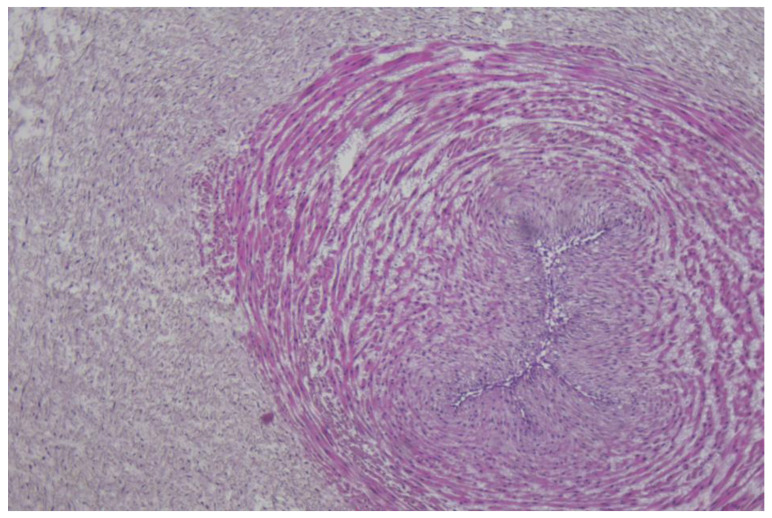
Histological aspect of umbilical cord section, lacking inflammatory reactions, H&E stain, ×100 magnification.

**Figure 3:**
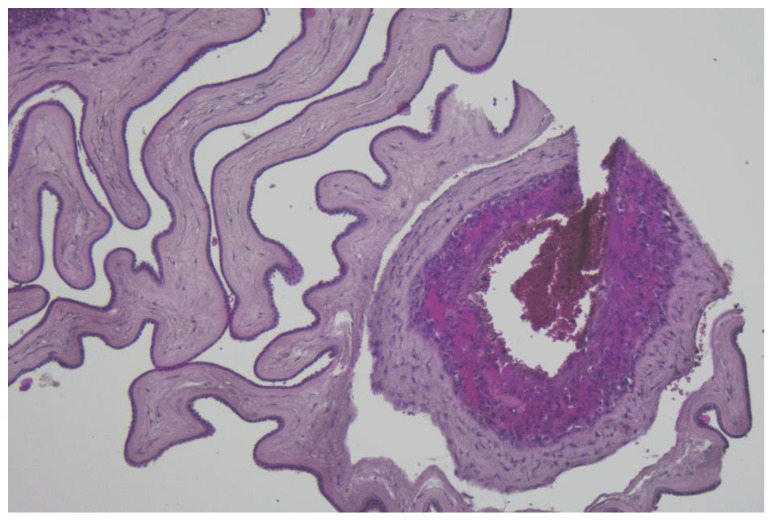
Amniotic membrane section, without inflammatory cells, H&E stain, ×100 magnification.

The immunohistochemistry analysis of the umbilical cord, amniotic membranes, and placental fragments from pregnant patients with COVID-19 failed to demonstrate positivity for the following markers: CD19, CD3, CD4, CD8, and CD56. These antibodies were used in order to find the inflammatory cells in the analyzed tissues as follows: CD19 for B cells, CD3 for T cells, CD4 for T helper cells, CD8 for cytotoxic T cells, and CD56 for NK cells. Tissue samples stained with hematoxylin and eosin, as well as immunohistochemical markings, are presented in Figure 1, Figure 2 and Figure 3.

## 4. Discussion

In this prospective study, we assessed the immunohistochemical changes in the umbilical cord, amniotic membranes, and placental fragments, the immunophenotyping alterations, and pregnancy outcomes in a cohort of patients with this SARS-CoV-2 infection. Personal history of pulmonary disease and smoking habit were more prevalent for pregnant patients with this viral infection. Regarding the patient’s symptomatology, anosmia, cough, ageusia, and fever were the most frequently encountered manifestations at admission.

Previous research revealed similar results in terms of risk factors for COVID-19 development and its clinical picture. For example, in a Mendelian randomization study by Yeung et al., on a large cohort of adult patients, the authors demonstrated that smoking increased the risk of developing COVID-19 (OR: 1.19, 95% CI: 1.11–1.27), as well as the disease severity [33]. Moreover, a recent study by Radzikowska et al., that evaluated different SARS-CoV-2 receptors in primary human cells and tissues, demonstrated that the airway epithelium in asthma and chronic obstructive pulmonary disease (COPD) had a gene signature that potentially can facilitate viral entry and internalization into cells [34].

Anosmia and ageusia are frequently encountered symptoms in COVID-19 patients [35], and a recent systematic review and meta-analysis confirmed our findings regarding the clinical manifestations of pregnant patients by citing cough, fever, fatigue, and anosmia/ageusia as the most commonly reported symptoms in the literature [36].

The univariate analysis of the main laboratory parameters revealed a significantly higher frequency of inflammatory syndrome (elevated C-reactive protein, procalcitonin, and ferritin) in the group of patients diagnosed with COVID-19. As expected, the inflammatory syndrome associated with COVID-19 was also outlined in various studies on pregnant patients [37,38,39]. Moreover, it was demonstrated that elevated serum levels of procalcitonin and ferritin were associated with severe forms of disease [40,41].

On the other hand, our results failed to indicate a statistically significant difference between the two groups regarding adverse pregnancy outcomes, such as preterm labor and neonatal intensive care unit admission. A multicentric cohort study by Oncel et al. revealed high rates of cesarean section (71.2%), prematurity (26.4%), and low-birthweight-infant rates (12.8%) for a cohort of patients with COVID-19 [42]. Another retrospective multicentric study indicated higher rates of NICU admission among offspring of symptomatic women [43].

We hypothesize that the small cohort of patients represents the main reason for our findings in contrast with the published data. Another explanation for these findings could be represented by the longer timeframes between SARS-CoV-2 infection and birth. Specifically, this type of viral infection acquired in the first trimester of pregnancy, when placentation occurs, could potentially have a greater impact on the development of ischemic placental disease that encompasses intrauterine growth restriction.

The subgroup analysis of pregnant patients using immunophenotyping indicated that patients with moderate–severe forms of COVID-19 had a significantly reduced population of lymphocytes, CD4+ T cells, CD8+ T cells (only numeric), and CD4+/CD8+ index. Additionally, the populations of B lymphocytes and natural killer (NK) cells were significantly diminished in the first subgroup. Several studies demonstrated that lymphopenia and decreased NK cells were associated with progression to severe forms of COVID-19 in pregnancy [44,45,46,47,48]. Moreover, it was demonstrated that the absolute lymphocyte count (ALC) (AUC = 0.80) and the neutrophil to lymphocyte ratio (NLR) (AUC = 0.86) were highly sensitive for progression to severe illness [46].

The lymphocytopenia could be due to viral attachment, immunological damage caused by proinflammatory cytokines, or extravasation of circulating lymphocytes into lung tissues [44]. In a prospective study by Wang et al., that evaluated the levels of peripheral lymphocyte subsets in 60 hospitalized COVID-19 patients, the authors demonstrated a decrease in total lymphocytes, CD4+ T, CD8+ T, B, and NK cells, especially in severe forms. Additionally, the authors showed a significant association between the inflammatory status of patients and decreased levels of CD8+ T cells and the CD4+/CD8+ ratio [49].

Several different mechanisms of programmed cell death, including pyroptosis, necroptosis, and PANoptosis of T cells, have been proposed as being responsible for lymphopenia. These mechanisms were demonstrated in a prospective study conducted by André et al. on 41 non-pregnant subjects who were infected with SARS-CoV-2 [50]. Moreover, up-regulation of proapoptotic proteins (B-cell lymphoma-2 protein-BCL-2, C-X-C motif chemokine ligand 10- CXCL10, and C-C motif chemokine ligand 2- CCL2) as well as interleukin 6 (IL-6) were presented as molecular alterations associated with lymphopenia in patients with this infection [51]. These mechanisms of lymphopenia could have an additive effect on the immunosuppressive state induced by progesterone in pregnancy.

Finally, our immunohistochemistry analysis of umbilical cords, amniotic membranes, and placental fragments retrieved in the third trimester of pregnancy failed to demonstrate positivity for CD19, CD3, CD4, CD8, and CD56 markers. In a recent study by Levitan et al., the authors evaluated 64 placentas using immunohistochemical staining for SARS-CoV-2 nucleocapsid protein, and found that none of the specimens were positive for this marker [52]. Another study that analyzed the immunohistochemical staining of placental specimens for various leukocytes revealed an increased CD68+ macrophage infiltration [53].

Resta et al. investigated the association between the symptoms’ severity and different placental histological characteristics using immunohistochemical investigations for CD4 + and CD8 + T lymphocytes, and CD68 + macrophages [26]. The authors did not observe significant differences between patients with mild or severe forms of COVID-19 and controls for CD4. CD8 expression was significantly higher in placentas from patients with severe forms compared with controls, while the CD68 expression was significantly different between the evaluated groups. Finally, Juttukonda et al. characterized the decidual immune response depending on the timing of infection during gestation, and demonstrated that for an infection acquired in the third trimester of pregnancy, decidual tissues presented significantly more macrophages (CD14+), NK cells (CD56+), and T cells (CD3+) [54]. For the infection acquired in the second trimester of pregnancy, only T cells (CD3+) were significantly more expressed in the evaluated placentas.

We hypothesize that a possible explanation for the lack of immunohistochemical positivity of the evaluated markers in the placenta, umbilical cord, and amniotic membranes could be represented by the fact that SARS-CoV-2 infection occurred in the third trimester of pregnancy for the patients enrolled in the study, and the timeframe from infection to birth was generally short for an intensive local immune response.

This study has the advantage of presenting the immunophenotyping and immunohistochemical findings from pregnant patients with acquired COVID-19 infection in the third trimester of pregnancy. However, the results outlined in this paper must be evaluated considering the following limitations: the small cohort of patients and a reduced timeframe for the prospective assessment.

Further studies, on larger cohorts of patients, could determine the extent of immunological decidual response in patients with various forms of COVID-19 and for different timings of infection during gestation.

## Figures and Tables

**Table 1:** Commercial antibodies for immunohistochemistry.

Product Number	Name	Species	Clone
IR65661-2	CD19	Mouse mAb	LE-CD19
IR50361-2	CD3	Mouse mAb	F7.2.38
IR64961-2	CD4	Mouse mAb	4B12
IR62361-2	CD8	Mouse mAb	C8/144B
IR62861-2	CD56	Mouse mAb	123C3

Table legend: mAB—monoclonal antibody.

**Table 2:** Univariate analysis of the clinical characteristics for the patients included in our study.

Patient’s Data		Group 1 (with COVID-19, *n* = 26 Patients)	Group 2 (without COVID-19, *n* = 26 Patients)	*p* Value
Demographics	Maternal age, years (mean and standard deviation)	29.73 ± 5.86	29.69 ± 6.74	0.98
	Medium (*n*/%)	Rural = 13 (50%) Urban = 13 (50%)	Rural = 11 (42.3%) Urban = 15 (57.7%)	0.39
Clinical parameters	BMI, kg/m^2^, (mean and standard deviation)	30.05 ± 5.99	29.93 ± 6.11	0.47
	Smoking (*n*/%)	Yes = 5 (19.2%)	Yes = 2 (7.7%)	0.037
	Pulmonary disease (*n*/%)	Yes = 4 (15.4%)	Yes = 0 (0%)	0.038
	Personal history of thrombosis (*n*/%)	Yes = 2 (7.7%)	Yes = 0 (0%)	0.14
	Diabetes (*n*/%)	Yes = 1 (3.8%)	Yes = 0 (0%)	0.31
	Thrombophilia (*n*/%)	Yes = 4 (15.4%)	Yes = 2 (7.7%)	0.38
	Lower limb varicose veins (*n*/%)	Yes = 2 (7.7%)	Yes = 1 (3.8%)	0.55

Table legend: BMI—body mass index.

**Table 3:** Univariate analysis of the paraclinical parameters at admission for patients included in our study.

Laboratory Parameters	Group 1 (with COVID-19, *n* = 26 Patients)	Group 2 (without COVID-19, *n* = 26 Patients)	*p* Value
Leucocytes/mm^3^ (mean, standard deviation)	9730 ± 3173.48	8900 ± 1839.83	0.57
Neutrophils, %, (mean, standard deviation)	76.48 ± 6.99	72.36 ± 6.80	0.11
Lymphocytes, %, (mean, standard deviation)	11.96 ± 6.46	13.2 ± 4.96	0.34
Monocytes, %, (mean, standard deviation)	5.70 ± 1.59	6.62 ± 0.98	0.22
Eosinophils, %, (mean, standard deviation)	0.93 ± 0.66	1.06 ± 0.60	0.35
Basophils, %, (mean, standard deviation)	0.98 ± 0.33	1.18 ± 0.37	0.12
Thrombocytes/mm^3^ (mean, standard deviation)	264,692.3 ± 124,452	208,200 ± 79,948.1	0.17
C-reactive protein, mg/dL (mean, standard deviation)	13.2 ± 7.35	1.47 ± 0.29	<0.001
Procalcitonin, ng/mL (mean, standard deviation)	5.7 ± 6.85	0.14 ± 0.06	<0.001
Glutamic-oxaloacetic transaminase (TGO), U/L (mean, standard deviation)	55.5 ± 6.82	39.4 ± 2.95	0.38
Glutamic pyruvic transaminase (TGP), U/L (mean, standard deviation)	63.73 ± 1.53	18.91 ± 17.44	0.43
Ferritin, ng/mL (mean, standard deviation)	620.05 ± 248.89	1344.58 ± 444.92	<0.001

Table legend: TGO—Glutamic-oxaloacetic transaminase, TGP—Glutamic pyruvic transaminase.

**Table 4:** Pregnancy outcomes for the two groups of patients included in the study.

Pregnancy Outcomes	Group 1 (with COVID-19, *n* = 26 Patients)	Group 2 (without COVID-19, *n* = 26 Patients)	OR and 95%CI	*p* Value
Gestational age, weeks (mean, standard deviation)	38.19 ± 1.91	38.53 ± 1.10	0.99 (0.998–1.001)	0.42
Birth weight, g (mean, standard deviation)	3151.92 ± 564.70	3189.23 ± 406.08	0.70 (0.46–1.06)	0.39
Apgar score 5 min (mean, standard deviation)	8.38 ± 0.63	8.88 ± 0.58	4.11 (1.211–13.974)	0.005
Mode of delivery (*n*/%)	Cesarean = 15 (57.7%) Vaginal = 11 (42.3%)	Cesarean = 17 (65.4%) Vaginal = 9 (34.6%)	1.12 (0.320–3.924)	0.56
Intrauterine growth restriction (*n*/%)	Yes = 4 (15.4%)	Yes = 2 (7.7%)	0.32 (0.03–3.12)	0.38
Preterm labor (*n*/%)	Yes = 5 (19.2%)	Yes = 2 (7.7%)	0.98 (0.96–1.00)	0.22
Neonatal intensive care unit admission (*n*/%)	Yes = 1 (3.8%)	Yes = 0 (0%)	1.01 (0.98–1.05)	0.31

Table legend: OR—odds ratio; CI—confidence interval.

**Table 5:** Univariate analysis of lymphocyte populations among the evaluated subgroups.

Parameters Evaluated	Moderate–Severe Form (Subgroup 1, *n* = 12 Patients)	Mild Form (Subgroup 2, *n* = 14 Patients)	*p* Value
Lymphocyte number/mm^3^ (mean, standard deviation)	746.0 ± 429.75	1927.85 ± 717.30	<0.001
Lymphocyte percentage	68.41 ± 6.76	79.21 ± 5.60	0.002
CD4+ T cells number/mm^3^ (mean, standard deviation)	531.08 ± 350.42	1538.78 ± 588.09	<0.001
CD4+ T cells percentage	32.25 ± 11.17	47.92 ± 8.85	0.003
CD8+ T cells number/mm^3^ (mean, standard deviation)	275 ± 248.85	950.71 ± 393.79	<0.001
CD8+ T cells percentage	30.16 ± 3.45	29.07 ± 7.16	0.31
CD4+/CD8+ index	1.12 ± 0.55	1.77 ± 0.63	0.005
B lymphocytes number/mm^3^ (mean, standard deviation)	101.08 ± 66.37	227.5 ± 141.77	0.004
B lymphocytes percentage	13.58 ± 3.67	11.14 ± 4.89	0.08
Natural killer cells number/mm^3^ (mean, standard deviation)	83.75 ± 28.96	134.85 ± 42.67	0.001
Natural killer cells percentage	12 ± 1.85	8.35 ± 4.74	0.02

## Data Availability

The data presented in this study are available on request from the corresponding author. The data are not publicly available due to local policies.
